# Supplementary material for: Heterogeneous biological membranes regulate protein partitioning via fluctuating diffusivity
Source: PNAS Nexus. 2023 Aug 3;2(8):pgad258. doi: 10.1093/pnasnexus/pgad258 (PMC10427746; doi:10.1093/pnasnexus/pgad258)
Supplement: pgad258_Supplementary_Data [file pgad258_supplementary_data.zip › PNASNEXUS-PNASNEXUS-2023-00127R-s02.pdf]

**Supporting Information for**
# Heterogeneous biological membranes regulate protein partitioning via fluctuating diffusivity

Ken Sakamoto, Takuma Akimoto, Mayu Muramatsu, Mark S. P. Sansom,
Ralf Metzler, and Eiji Yamamoto

Paste corresponding author: Eiji Yamamoto
Email: eiji.yamamoto@sd.keio.ac.jp

**This PDF file includes:**

Figures S1 to S4
Legends for Movies S1

**Other supporting materials for this manuscript include the following:**

Movies S1

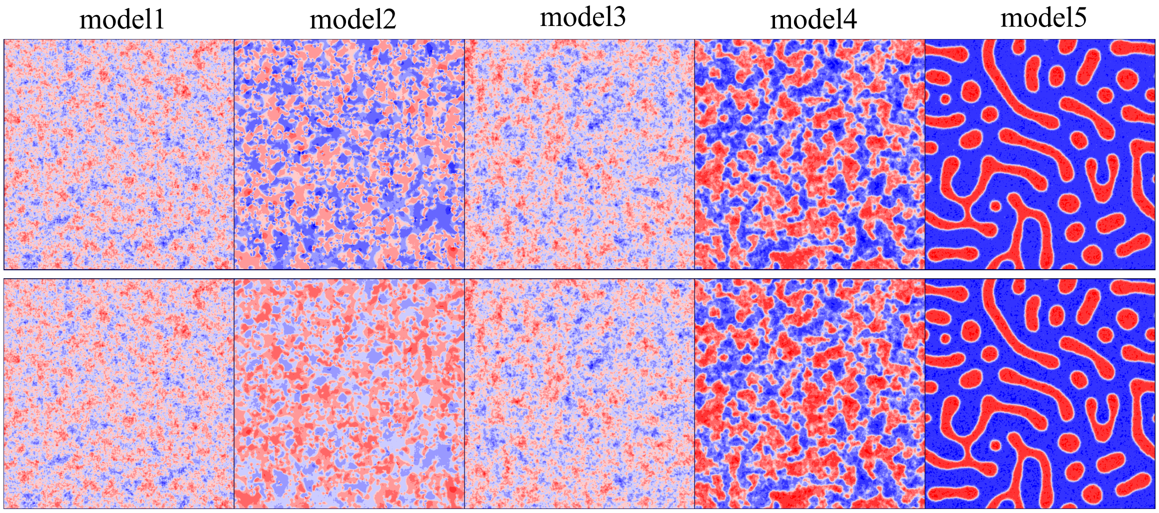


Fig. S1. The upper and lower figures show snapshots of order parameter *c* distribution at the equilibrium state and those at 0.1 ms after the equilibrium state, respectively.

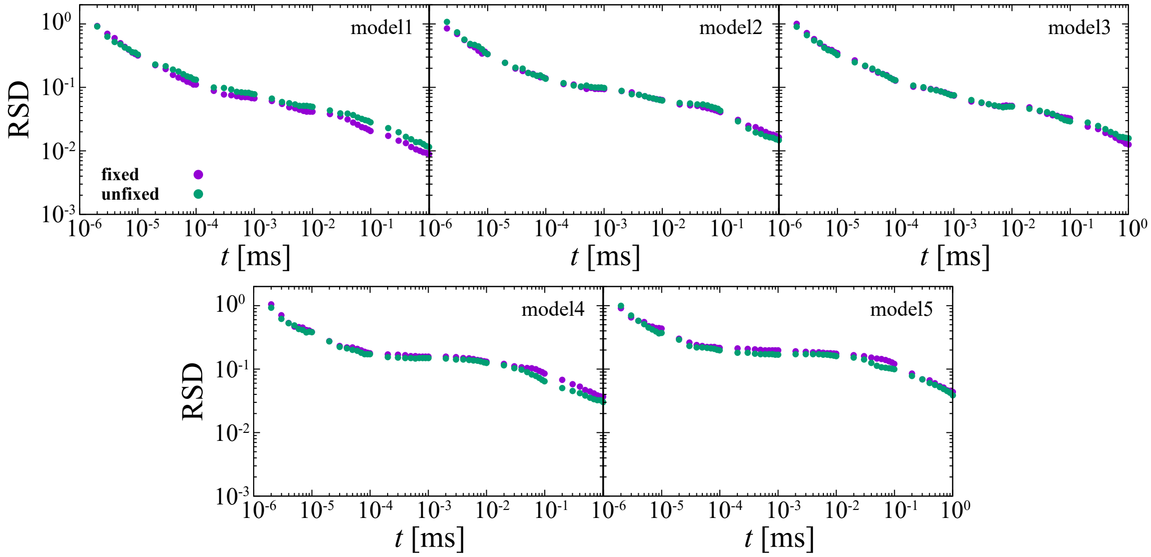


Fig. S2. RSDs of TAMSDs for 100 trajectories using fixed field or time-varying field.

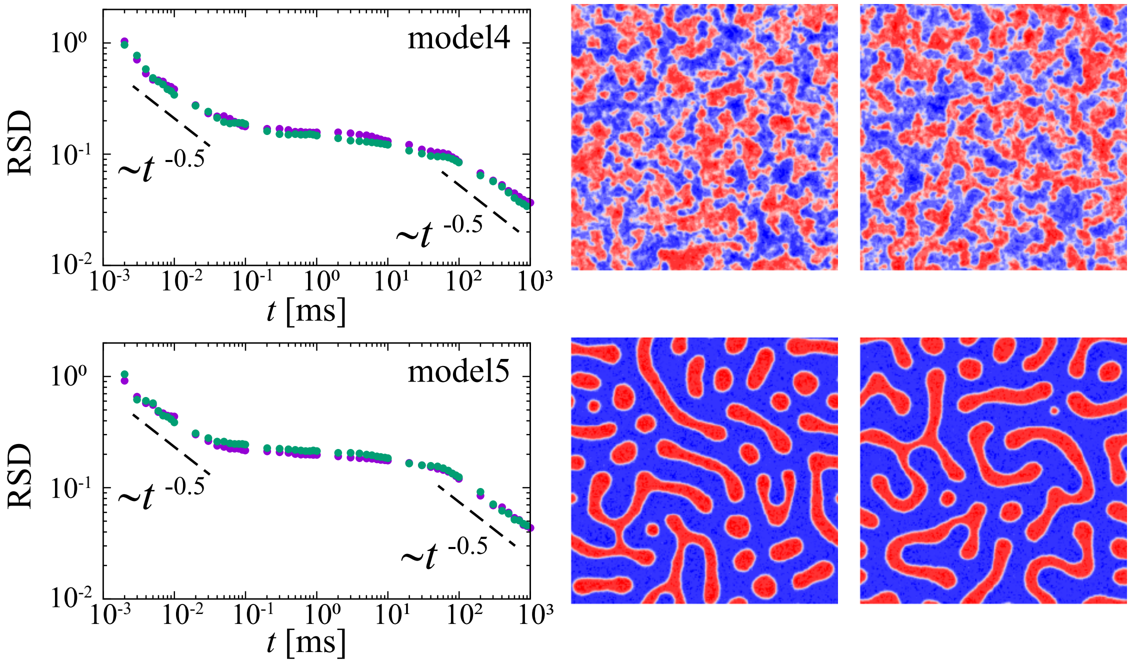


Fig. S3. RSD of TAMSDs for 100 trajectories obtained from single-particle simulations. Different colored points represent the simulation results of using different phase-separated field patterns. There is no dependence on the field patterns.

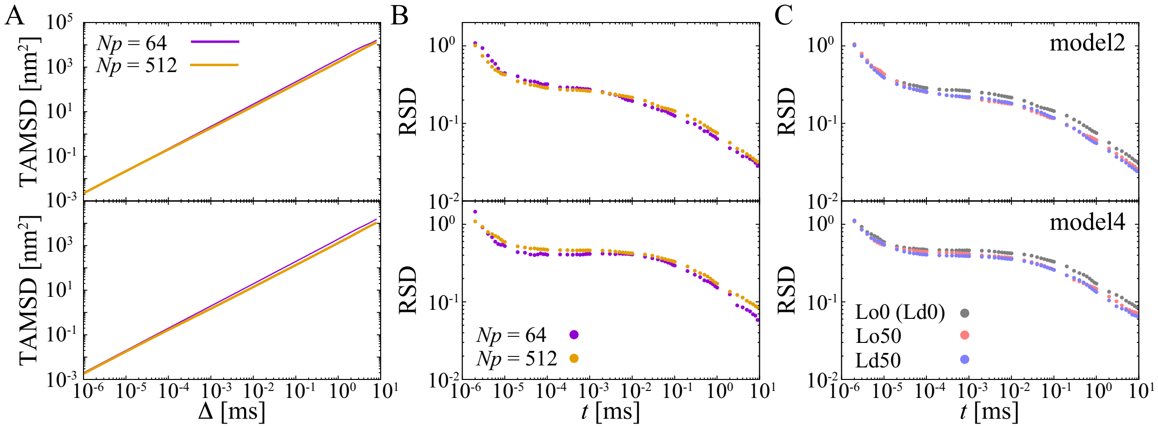


Fig. S4. (A) TAMSD and (B) RSD with $\varepsilon$ = 2.0 and $L_o0(L_d0)$. (C) RSD with $\varepsilon$ = 2.0 and $N_p$ = 512. Upper and lower figures show the results for model2 and model4, respectively.

Movie S1. Simulation of $N_p$ = 512 with $\varepsilon$ = 2.0. Simulation time is for 1.2 ms. Red and blue colored regions represent $L_o$ and $L_d$ domains, respectively. Molecules in $L_o$ and $L_d$ domains are colored cyan and magenta, respectively.
